# Genome–wide association study for risk taking propensity indicates shared pathways with body mass index

**DOI:** 10.1038/s42003-018-0042-6

**Published:** 2018-05-03

**Authors:** Emma A. D. Clifton, John R. B. Perry, Fumiaki Imamura, Luca A. Lotta, Soren Brage, Nita G. Forouhi, Simon J. Griffin, Nicholas J. Wareham, Ken K. Ong, Felix R. Day

**Affiliations:** 1 MRC Epidemiology Unit, University of Cambridge School of Clinical Medicine, Institute of Metabolic Science, Cambridge, CB2 0SL UK; 2 Department of Public Health and Primary Care, Institute of Public Health, University of Cambridge, Cambridge, CB2 0SR UK

## Abstract

Risk-taking propensity is a trait of significant public health relevance but few specific genetic factors are known. Here we perform a genome-wide association study of self-reported risk-taking propensity among 436,236 white European UK Biobank study participants. We identify genome-wide associations at 26 loci (*P* < 5 × 10^−8^), 24 of which are novel, implicating genes enriched in the GABA and GABA receptor pathways. Modelling the relationship between risk-taking propensity and body mass index (BMI) using Mendelian randomisation shows a positive association (0.25 approximate SDs of BMI (SE: 0.06); *P* = 6.7 × 10^−5^). The impact of individual SNPs is heterogeneous, indicating a complex relationship arising from multiple shared pathways. We identify positive genetic correlations between risk-taking and waist-hip ratio, childhood obesity, ever smoking, attention-deficit hyperactivity disorder, bipolar disorder and schizophrenia, alongside a negative correlation with women’s age at first birth. These findings highlight that behavioural pathways involved in risk-taking propensity may play a role in obesity, smoking and psychiatric disorders.

## Abstract (web summary)

Emma Clifton et al. report a genome-wide association study of risk taking propensity amongst UK Biobank participants. They identify 26 loci, 24 of which are novel, and use Mendelian randomisation analysis to explore the relationship between risk-taking propensity and BMI.

## Introduction

Risk-taking propensity describes a tendency to engage in reward-seeking actions despite the possibility of negative consequences^1^. While risk-taking peaks during adolescence, individual differences show longitudinal stability and risk-taking propensity is considered a stable trait^2,3^ representing an established risk factor for health-related behaviours including smoking, alcohol use and binge-eating^4–7^. Results from experimental and population-based studies have generated interest in the possible association between risk-taking propensity and body mass index (BMI)^8,9^.

Risk-taking, measured using varied methods, has been associated with obesity in experimental and cross-sectional observational studies^8^. Compared to their normal weight peers, adolescents with a BMI >99th percentile for their age and sex exhibit greater odds of ever having smoked and having used drugs or alcohol before their last sexual encounter^10^. Other evidence suggests that obese individuals are more likely to neglect long-term outcomes in decision-making^11^. Indeed, risk-taking propensity has been associated with impulsivity^12^, a trait characterised by a tendency to act without adequate forethought and associated with obesity^13–16^, weight gain^9^ and binge-eating^17^.

Studies of attention-deficit hyperactivity disorder (ADHD) support the existence of an association between impulsivity and BMI^18^. Those with ADHD are more likely to take risks^19,20^, binge-eat^21^ and be obese^22^ than those without ADHD. Further, obese individuals with ADHD exhibit more severely disordered eating patterns than obese individuals without ADHD^23^. Weight management among ADHD sufferers is improved by pharmacological ADHD treatments^22^. In one study, psychostimulant ADHD drug treatment decreased binge-eating, impulsive food selection and BMI among a group of severely obese adults recently diagnosed with ADHD^24^.

While studies suggest an association between risk-taking propensity and obesity, evidence of causality is lacking. ADHD-like symptoms observed in ~80% of homozygous carriers of *MC4R* mutations, resulting in severe obesity, suggests the possibility of reverse causality or shared pathways^25^. An informative approach to exploring the causal relationship between risk-taking and BMI is Mendelian randomisation (MR) using genetic variants associated with risk-taking as instrumental variables. Heritability estimates for risk-taking range between 0 and 55%, indicating that genetic approaches may be possible^2,26^.

Previous genome wide association studies (GWAS) of risk-related behaviours have been reported. Among 125,667 adults from UK Biobank, 38 loci were identified for age at first sexual intercourse^27^. One SNP, intronic to *CADM2* (rs57401290), has subsequently been associated with risk-taking propensity assessed by the question: *Overall*, *do you feel comfortable or uncomfortable taking risks?* in an independent sample of 140,487 participants from 23andMe using a ‘phenome-scan’ for associations between *CADM2* and personality traits (rs1865251; *r*^2^ with rs57401290 = 0.78)^28^. A GWAS of risk-taking propensity has been conducted among 116,225 UK Biobank participants based on the question: *Would you describe yourself as someone who takes risks?* The study identified two genome-wide significant loci, one within *CADM2* and one within the human leukocyte antigen (HLA) region on chromosome 6. Genetic correlations between risk-taking and schizophrenia, bipolar disorder (BPD), ADHD, post-traumatic stress disorder, smoking and obesity were also identified^29^.

To identify specific genetic variants robustly associated with risk-taking propensity, we perform a GWAS among 436,236 white Europeans and link the findings to other genome-wide results for gene expression, as well as for mental health and other outcomes. We identify genome-wide associations at 26 loci. MR modelling suggests a complex relationship between risk-taking and BMI, resulting from multiple shared pathways. Genetic correlations highlight links between risk-taking propensity and obesity, smoking and psychiatric disorders.

## Results

### Population characteristics

The GWAS sample comprised 436,236 UK Biobank (UKB) participants of white European descent. The mean age of participants at enrolment was 56.8 years (SD = 8.0) and 54.1% were women. Of this sample, 113,882 (26.1%) responded ‘Yes’ and 322,354 (73.9%) responded ‘No’ to the question: *Would you describe yourself as someone who takes risks?*

Risk-taking propensity was recorded on repeat occasions in a sub-set of participants. Repeat measures multivariate analysis of variance showed that risk-taking propensity at each time point was very strongly associated with risk-taking propensity at later time points (*P* = 6.02 × 10^−6^). Of all participants in UK Biobank with repeated measures, including those of non-European ancestry, 16,385 out of 19,006 (86.2%) recorded the same response between baseline and their first follow-up, 10,102 of 12,084 (83.6%) recorded the same response between baseline and their second follow-up and 3300 of 3816 (86.5%) recorded the same response between their first and second follow-ups. ‘Risk-takers’ were more likely to be male, younger and have a higher BMI (Table 1). Compared to non-risk-takers, they were also more likely to report specific risk-taking behaviours, such as ever having smoked or experienced substance addiction. Further, among women who reported having had children, risk-takers gave birth to their first child at a younger age. We did not find any association with clinical eating disorders or schizophrenia, both of which were reported in very small numbers of individuals in this sample; but there was a positive association between risk-taking and depression. Surprisingly, risk-takers reported older age at leaving education. However, the SD for this outcome variable was also significantly larger among the risk-takers than the non-risk-takers (Levene’s test *P* < 1 × 10^−8^) indicating more variability in those identifying as risk-takers.

**Table 1:** Descriptive information by UKB participants’ answers to the question: *Would you describe yourself as someone who takes risks?*

	Yes (*n* = 113,882)	No (*n* = 322,354)	*P* value
Female	39.5%	59.2%	<1 × 10^−200^
Age	55.8 (8.2)	57.1 (7.9)	<1 × 10^−200^
BMI (kg/m^2^)^a^	27.7 (4.7)	27.3 (4.8)	3.1 × 10^−81^
Age at first birth^b^	25.2 (4.9)	25.4 (4.5)	1.1 × 10^−20^
Ever smoked^a^	53.3%	43.3%	<1 × 10^−200^
Alcohol frequency (self-report)^a^	Median—‘three or four times a week’	Median—‘once or twice a week’	<1 × 10^−200^
Drug addiction (self-report)^a^	0.33%	0.11%	4.4 × 10^−32^
Any eating disorder (self-report)^a^	0.08%	0.07%	0.22
Schizophrenia (self-report)^a^	0.12%	0.11%	0.17
Depression (self-report)^a^	6.18%	5.96%	2.3 × 10^−14^
Age completed education^a^	16.7 (2.4)	16.6 (2.1)	6.5 × 10^−6^

*BMI* body mass index

Values are mean (SD) or %, except for alcohol frequency where the responses were on a six point scale ranging from ‘Never’ to ‘Daily or almost daily’

^a^ Age- and sex-adjusted models used to calculate the *P* value in a regression model—linear for continuous phenotypes, logistic for binary phenotypes and ordered categorical for alcohol frequency

^b^ Data for women only, the *P* value is from a model with only age adjustm`ent

### Genomic loci

**Fig. 1:**
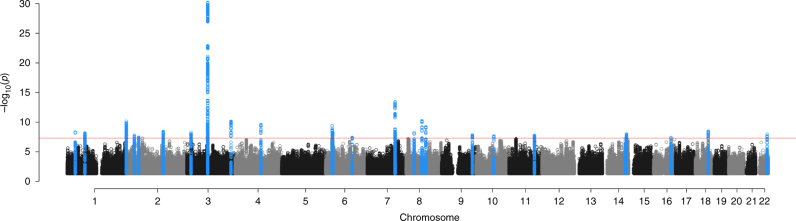
Manhattan plot of the GWAS of risk-taking propensity . The plot illustrates the results of the GWAS of 436,236 participants of white European descent in UK Biobank. Negative log-transformed *P* values for each SNP (*y* axis) are plotted by chromosomal position (*x* axis). The red-dashed line indicates the threshold for statistical significance (*P* = 5 × 10^−8^). The blue dots indicate SNPs within a 1-Mb region of a genome-wide significant signal

Chip heritability for risk-taking propensity in UKB was estimated to be 0.084 (SE = 0.002). This estimate is on the observed scale, precluding comparison to other estimates^30^. Twenty-six loci were associated with risk-taking propensity at genome-wide significance (*P* < 5 × 10^−8^) (Fig. 1; Table 2). The odds of self-reported risk-taking propensity ranged from 1.022 to 1.049 per allele. The strongest signal, rs6762267 lies intronic in *CADM2*. This SNP is in high linkage disequilibrium  (LD) with both SNPs previously reported in association with risk-taking, which were also intronic in *CADM2* (rs57401290: *r*^2^ = 0.78; rs13084531: *r*^2^ = 0.49)^27,29^. Other correlated *CADM2* variants have also previously been reported in association with BMI (rs13078960: *r*^2^ = 0.21)^31^, educational attainment (rs62263923: *r*^2^ = 0.27; rs55686445: *r*^2^ = 0.27)^32,33^ and alcohol consumption (rs9841829: *r*^2^ = 0.49)^34^. The second strongest signal that we identified for risk-taking propensity, rs727644 lies intronic in *FOXP2*, which has previously been associated with age at first birth in women (rs10953766: *r*^2^ = 0.14)^35^. We observed a low intercept value for the LD score regression GWAS (1.02, SE: 0.01), indicating that the vast majority of test statistic inflation (lambda genomic control (GC) = 1.37) is due to polygenicity rather than population structure.

**Table 2:** Twenty-six genome-wide significant loci for risk-taking propensity from the UK Biobank study

Variant	Chr	Pos	Implicated gene	SNP location	Alleles^a^	Allele freq.^b^	OR	95% CI	*P* value	Gene-associated disorders and phenotypes
rs6762267	3	85513115	*CADM2* ^N,E^	Intronic	C/A	0.38	1.049	1.041–1.058	6.60 × 10^−31^	—
rs727644	7	114109349	*FOXP2* ^N,E^	Intronic	G/A	0.60	1.031	1.023–1.040	4.00 × 10^−14^	Speech and language disorder 1
rs62519827	8	65481947	*CYP7B1* ^E,M^	Intergenic	T/C	0.89	1.042	1.029–1.055	6.00 × 10^−11^	Spastic paraplegia
rs9841382	3	181408124	*SOX2-OT* ^N^	Intronic	C/T	0.14	1.038	1.026–1.049	7.10 × 10^−11^	CNS abnormalities; development delay
rs58560561	1	243537729	*SDCCAG8* ^N,E^	Intronic	G/T	0.65	1.028	1.019–1.036	7.20 × 10^−11^	Educational attainment; Bardet–Biedl syndrome
rs992493	4	106180264	*TET2* ^N^	Intronic	T/C	0.19	1.033	1.023–1.043	2.50 × 10^−10^	—
rs6923811	6	27289776	*POM121L2* ^N^	Intergenic	T/C	0.68	1.027	1.019–1.036	3.90 × 10^−10^	Autistic spectrum disorder
rs7817124	8	81404008	*ZBTB10* ^N^	Intronic	C/G	0.24	1.030	1.020–1.039	6.10 × 10^−10^	—
rs4801000	18	53456943	*TCF4* ^N^	Intergenic	G/A	0.34	1.025	1.017–1.034	3.40 × 10^−9^	Schizophrenia
rs4653015	1	33776431	*ZNF362* ^E^	Intergenic	T/C	0.26	1.027	1.018–1.037	3.80 × 10^−9^	—
rs12476923	2	145830053	*DKFZp686O1327* ^N^	Intronic	A/C	0.34	1.025	1.017–1.034	4.70 × 10^−9^	—
rs283914	3	17330649	*TBC1D5* ^N,E^	Intronic	T/C	0.53	1.024	1.016–1.032	5.30 × 10^−9^	Schizophrenia
rs4233093	1	73446245	*NEGR1* ^N^	Intergenic	A/G	0.52	1.024	1.016–1.032	5.30 × 10^−9^	Neuronal growth
rs7829912	8	33479228	*DUSP26* ^N^	Intergenic	T/C	0.56	1.024	1.016–1.032	5.90 × 10^−9^	—
rs3117340	6	29210596	*OR14J1* ^N^	Intergenic	G/T	0.62	1.024	1.016–1.033	7.00 × 10^−9^	Autistic spectrum disorder; sensory experience
rs1381287	14	98597552	*RP11-61O1.1* ^N,E^	Intergenic	T/C	0.46	1.023	1.015–1.032	9.90 × 10^−9^	—
rs28520003	22	46411969	*LINC00899* ^E^	Intergenic	G/A	0.69	1.025	1.016–1.034	1.10 × 10^−8^	—
rs12115650	9	126367705	*DENND1A* ^N^	Intronic	G/A	0.72	1.026	1.017–1.035	1.50 × 10^−8^	—
rs11226319	11	104221573	*PDGFD* ^N^	Intergenic	A/G	0.16	1.032	1.021–1.043	1.50 × 10^−8^	Neocortical development
rs1358391	7	115111838	*SNORA25* ^N^	Intergenic	G/T	0.51	1.023	1.015–1.031	1.50 × 10^−8^	—
rs12617392	2	27336827	*CGREF1* ^N,E^	Intronic	C/A	0.56	1.023	1.015–1.031	1.80 × 10^−8^	—
rs542883	2	45143382	*SIX3* ^N,E^	Intergenic	C/G	0.56	1.023	1.015–1.031	2.20 × 10^−8^	Holoprosencephaly
rs10823791	10	73338334	*CDH23* ^N^	Intronic	T/A	0.40	1.023	1.015–1.031	3.60 × 10^−8^	Usher syndrome; profound deafness
rs34905321	6	109131107	*ARMC2* ^N^	Intergenic	T/C	0.57	1.022	1.014–1.031	3.90 × 10^−8^	—
rs891124	16	71440756	*CALB2* ^N^	Intergenic	T/C	0.71	1.024	1.016–1.033	4.10 × 10^−8^	—
rs35914833	14	94182383	*PRIMA1* ^N^	Intergenic	T/C	0.68	1.024	1.015–1.033	5.00 × 10^−8^	—

*OR* odds ratio, *N* nearest gene, *E* eQTL, *M* missense

^a^ Effect allele/other allele

^b^ Effect allele frequency

As a sensitivity analysis, we repeated the GWAS of risk-taking using data measured at the two later time points. A total of 18,768 individuals of European ancestry reported risk-taking at the first follow-up assessment. At this time point, 22 of 26 SNPs showed directional consistency with our baseline GWAS (binomial test for directional consistency between time points: *P* = 0.0005). A total of 11,887 individuals reported risk-taking at the second follow-up assessment. At this time point, 19 of 26 SNPs showed directional consistency with our baseline results (binomial test: *P* = 0.029).

In addition, while true replication of our results was not possible due to lack of available, independent data, we conducted a GWAS of ‘ever smoking’ in UK Biobank for the purpose of looking up genome-wide significant SNPs for risk-taking. The sample contained 207,229 ever smokers (46%) and 243,177 never smokers. The results are presented in Supplementary Table 1. Eleven of the 26 risk-taking SNPs showed Bonferroni significant associations with ever smoking (corrected for 26 tests: *P* < 0.0019) and 13 reached nominal significance (*P* < 0.05). All of these showed directionally consistent associations between risk-taking and ever smoking. In total, 21 of the 26 SNPs were directionally consistent.

Other notable association signals include rs58560561 within *SDCCAG8*, which has been reported in association with educational attainment (rs2992632: *r*^2^ = 0.76)[32] ; rs6923811 near *POM121L2* and rs3117340 near *OR14J1*, which have both been reported in association with autistic spectrum disorder (rs141342723: *r*^2^ = 0.13; rs115329265: *r*^2^ = 0.24, respectively)^36^; and rs4801000 near *TCF4* (rs9636107: *r*^2^ = 0.46) and rs283914 within *TBC1D5* (rs4330281: *r*^2^ = 0.58), which have been reported in association with schizophrenia^37^. In addition, *NEGR1* has previously been reported in association with BMI^31^, although our signal appears to be independent of that reported signal (rs3101336; *r*^2^ with our signal (rs4233093) = 0.02).

Several of the genes that co-locate with risk-taking signals are reported to be mutated in rare disorders of central nervous system (CNS) functioning and neuro-developmental delay. For example, *CDH23* is mutated in Usher syndrome, characterised by profound deafness^38^, *CYP7B1* is mutated in a rare form of spastic paraplegia^39^, *SIX3* is mutated in holoprosencephaly resulting in major mental retardation^40^, and mutations in *FOXP2* are associated with speech and language disorder 1^41^. Further, mutations in *SOX2-OT* are associated with CNS abnormalities and neuro-developmental delay^42^ and mutations in *SDCCAG8* are associated with Bardet–Biedl Syndrome, features of which include obesity and neuro-developmental delay^43^. Other signals co-localise near genes that regulate CNS or sensory neural function. These include, *NEGR1* which is involved in neuronal growth^44^, *OR14J1*, which is involved in sensory experience^45^, and *PDGFD*, which is involved in human neocortical development^46^. One lead SNP (rs62519827) is in high LD (*r*^2^ = 0.98) with a missense variant (rs62519835) in *BHLHE22*, which encodes a transcription factor involved in neuronal differentiation and is also an eQTL for *CYP7B1*.

Four identified loci showed genome-wide significant associations with BMI (Table 3), including two novel signals for BMI (rs891124, which is an eQTL for *CALB2* and rs35914833 at *PRIMA1*). These loci were derived from a combination of UK Biobank and the GIANT consortium data and have not been reported in any previous BMI GWAS studies. Signals at *CADM2* and *ZBTB10* have previously been associated with BMI^31^. However, the four signals did not show directionally consistent associations. The risk-increasing variants at *CADM2*, *CALB2* and *PRIMA1* were associated with higher BMI, while the risk-increasing variant at *ZBTB10* was associated with lower BMI. Signals at *CALB2*, *ZBTB10* and *PRIMA1* showed nominally significant associations (*P* < 0.05) with TV snacking, skipping breakfast and daily energy intake, respectively (Table 3). None of these loci were associated with emotional eating (EE), uncontrolled eating (UE) or cognitive restraint (CR) in the Fenland cohort (all *P* > 0.10) (Supplementary Table 2).

**Table 3:** Associations between the four risk-taking loci that were genome-wide significant signals for BMI and  diet-related traits

Variant	Implicated gene	BMI		TV snacking		Home-cooked meals		Skipping breakfast		Energy (kcal/day)	
		Beta (SE)	*P* value	Beta (SE)	*P* value	Beta (SE)	*P* value	Beta (SE)	*P* value	Beta (SE)	*P* value
rs891124	*CALB2*	0.01 (0.002)	3.5 × 10^−10^	0.12 (0.05)	0.02*	0.04 (0.03)	0.21	0.01 (0.03)	0.86	3.86 (11.0)	0.73
rs35914833	*PRIMA1*	0.02 (0.002)	5.3 × 10^−14^	−0.05 (0.05)	0.34	−0.03 (0.03)	0.33	−0.04 (0.03)	0.20	30.3 (11.0)	0.01*
rs6762267	*CADM2*	0.02 (0.002)	1.7 × 10^−15^	0.09 (0.05)	0.07	0.02 (0.03)	0.45	−0.03 (0.03)	0.36	12.3 (10.2)	0.23
rs7817124	*ZBTB10*	−0.01 (0.002)	1.8 × 10^−9^	0.09 (0.06)	0.10	−0.03 (0.03)	0.36	0.08 (0.03)	0.02*	12.4 (11.5)	0.28

SNPs were aligned to the risk-taking propensity-increasing allele. Effect estimates (beta and SE) were derived from linear or logistic regressions of the variant to the named trait, adjusted for age and sex. BMI was a continuous outcome standardised within the BMI meta-analysis. TV snacking was coded: 0 never/rarely; 1 occasionally/ usually/ always; skipping breakfast was coded: 0 skips breakfast <2 times a week; 1 skips breakfast ≥2 times a week; home-cooked food was coded: 0:>5 meals a week home-cooked, 1:<5 meals a week are home-cooked

*Nominally significant (*P* < 0.05)

### Risk-taking to BMI, dietary patterns and eating behaviour

Using results from the present GWAS and an unpublished meta-analysis of BMI involving 772,825 individuals from GIANT and UK Biobank, we conducted a bi-directional MR analysis of risk-taking and BMI. In an inverse-weighted variance (IVW) model, genetically predicted risk-taking propensity predicted higher BMI (0.25 approximate SDs of BMI (SE = 0.06); *P* = 6.7 × 10^−5^), while genetically predicted BMI did not predict risk-taking propensity (*P* = 0.23) (Table 4).

**Table 4:** Mendelian randomisation analyses of BMI to risk-taking and risk-taking to BMI

Analysis	Beta (SE)	*P* value
*Risk-taking propensity to BMI*		
Conventional MR (IVW)	0.251 (0.063)	6.7 × 10^−5^
MR Egger	0.885 (0.985)	0.37
Weighted median MR	0.091 (0.121)	0.45
*BMI to risk-taking propensity*		
Conventional MR (IVW)	0.004 (0.004)	0.23
MR Egger	0.002 (0.017)	0.88
Weighted median MR	−0.008 (0.007)	0.26
*Between SNP heterogeneity*	Not applicable	9.9 × 10^−8^

*BMI* body mass index, *MR* mendelian randomisation, *IVW* inverse-weighted variance

MR Egger intercept was not significant

**Fig. 2:**
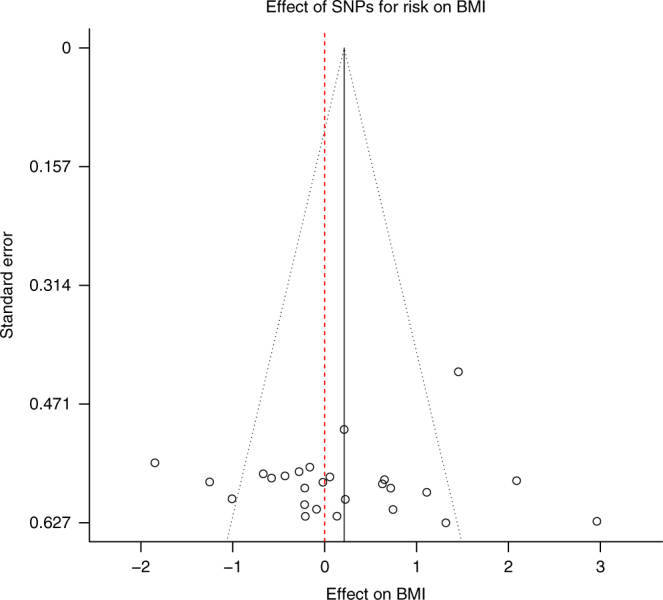
Effect of genome-wide significant SNPs for risk-taking on BMI. Each data point represents one of the 26 risk-associated SNPs. The SNP-specific MR estimate for the association of risk-taking with BMI (*x* axis) is plotted against the SE (*y* axis). The summary estimate is marked by the solid black line. The grey-dotted lines, originating from the summary estimate, represent 95% confidence limits. The red-dotted line indicates the null

There was a high level of between SNP heterogeneity in this analysis (*P* = 9.9 × 10^−8^), with individual risk-taking SNPs showing strong associations with either higher or lower BMI (Fig. 2). We performed a leave-one-out analysis, whereby we repeated the MR analysis of risk-taking to BMI 26 times with each of the genome-wide significant SNPs for risk-taking removed in turn. The results suggested that all four of the individually genome-wide significant SNPs had a substantial effect on the heterogeneity of the data (Supplementary Fig. 1). We performed a further MR analysis of risk-taking to BMI excluding the four risk-taking SNPs that were also genome-wide significant for BMI, and found no association between risk-taking propensity and BMI (beta from IVW MR = 0.01 (SE = 0.07); *P* = 0.91) or evidence of heterogeneity (*P* = 0.24). Similarly, a random effects IVW MR model, combining the estimates calculated when treating each risk-taking associated SNP as an individual instrument, also provided no evidence for an overall causal relationship between risk-taking and BMI (Supplementary Fig. 2).

In the Fenland cohort study, we performed a polygenic risk score (PRS) analysis. Genetically predicted risk-taking propensity showed positive associations with emotional eating in men, after adjustment for multiple testing, and nominally significant positive associations with total daily calorie, fat and protein intake in the combined cohort of men and women (Table 5).

**Table 5:** Polygenic risk score for risk-taking propensity (created using summary statistics from UKB) related to diet and eating behaviours in the Fenland study

Variable	Total (*N*)	Effect (95% CI)	*r* ^2^	*P* value
*All participants—nutrient intake*				
Energy (kcal/day)	8981	803.5 (140.1, 1466.8)	0.042	0.02*
Total fat (g/day)^a^	8981	0.52 (0.12, 0.92)	0.042	0.01*
Fruit and vegetables (g/day)^a^	8844	0.46 (−0.07, 0.99)	0.044	0.09
Protein (g/day)^a^	8981	0.36 (0.06, 0.66)	0.010	0.02*
Fibre (g/day)^a^	8981	0.28 (−0.10, 0.66)	0.005	0.15
Carbohydrates (g/day)^a^	8981	0.25 (−0.10, 0.60)	0.028	0.16
*Men only—eating behaviours*				
Emotional eating	1646	94.6 (35.7, 153.6)	0.007	0.002**
Cognitive restraint	1646	−2.62 (−48.0, 42.7)	0.005	0.91
Uncontrolled eating	1646	32.0 (−9.3, 73.3)	0.019	0.13
*Women only—eating behaviours*				
Emotional eating	1869	−21.2 (−82.7, 40.6)	0.002	0.50
Cognitive restraint	1869	−21.2 (−63.3, 20.8)	0.005	0.32
Uncontrolled eating	1869	14.8 (−24.0, 53.6)	0.013	0.45
*All participants—food-related behaviours*	*OR (95% CI)*			
TV snacking^b^	4414	1.03 (0.99, 1.06)	—	0.46
Skipping breakfast^b^	11,441	1.05 (1.02, 1.07)	—	0.03*
Home-cooked food^b^	11,439	0.99 (0.97, 1.01)	—	0.59

All models were linear or logistic regressions of the PRS for risk-taking to the variable, adjusted for age and sex. Sex-stratified models were only adjusted for age

TV snacking was coded: 0 never/rarely; 1 occasionally /usually/always; skipping breakfast was coded: 0 < 2 times a week; 1 ≥ 2 times a week; home-cooked food was coded: 0; > 5 meals a week home-cooked, 1; < 5 meals a week are home-cooked

*Nominally significant (*P* < 0.05)

**Bonferroni significant after adjustment for 15 tests (*P* < 0.003)

^a^ Log-transformed

^b^ Logistic regression

The ranges of the eating behaviours were as follows: CR: 0–100; UE: 0–96.3; EE: 0–100. The food-related behaviour variables were initially ordered categorical variables. However, their distributions were markedly non-normal. To account for this, they were dichotomised and logistic regression was performed. In all cases, the category containing the majority of participants was split from the rest of the sample. This was designed to increase the sample size of the comparison group and maximise the power of the analyses. The analysis revealed a nominally significant association between genetic risk-taking propensity and the higher odds of skipping breakfast more than twice a week (odds ratio (95% confidence interval) = 1.05 (1.02, 1.07)). No associations were observed between the risk-taking PRS and UE, CR or total daily fibre, fruit and vegetable or carbohydrate intake. Genetic risk-taking propensity did not predict the odds of eating home-cooked meals or snacking front of the television (Table 5).

### Tissues and pathways associated with risk-taking

**Fig. 3:**
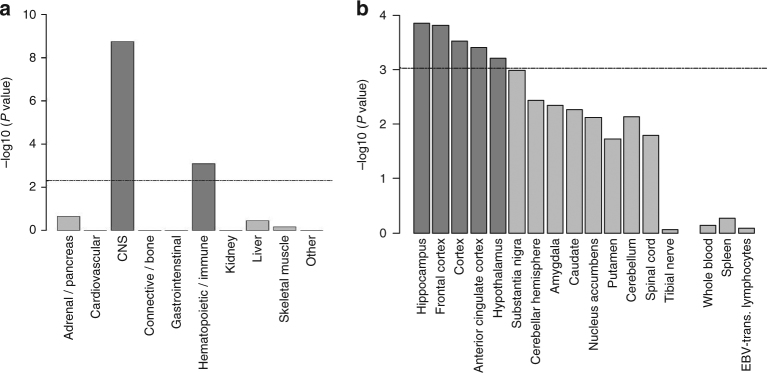
Tissue enrichment for risk-taking associated loci. **a** When tissues and cells are grouped together, GTEx analysis shows that genes within risk-taking loci are enriched for expression in the central nervous system (CNS) and hematopoietic/immune system. The dotted line indicates the threshold for statistically significant enrichment, established using the Bonferroni-corrected *P* value of partitioned heritability calculated by stratified LD score regression. **b** GTEx analysis shows enriched expression of genes within risk-taking loci in particular brain regions. The dotted line indicates the threshold for statistically significant enrichment, established using the Bonferroni-corrected *P* value of partitioned heritability calculated by stratified LD score regression

Tissue enrichment analysis using the GTEx database indicated that genes collocated with risk-taking variants were enriched for expression in the CNS (*P* = 1.80 × 10^−9^) and immune system (*P* = 8.20 × 10^−4^) (Fig. 3a). Of specific CNS tissues, the hippocampus, frontal cortex, cortex, anterior cingulate cortex and hypothalamus showed enrichment of expression after correction for multiple testing (Fig. 3b).

To identify mechanisms that may influence risk-taking propensity, we performed a systematic test of all annotated biological pathways for enrichment of genes located near risk-taking propensity-associated variants using MAGENTA. Two overlapping pathways were associated with risk-taking: the GABA pathway (false discovery rate (FDR) based on 75% cutoff = 0.006) and GABA receptor pathway (FDR based on 75% cutoff = 0.04). Full MAGENTA output is provided (Supplementary Data 1). Overlap between the two identified pathways is depicted in Supplementary Fig. 3.

### Genetic correlations

**Fig. 4:**
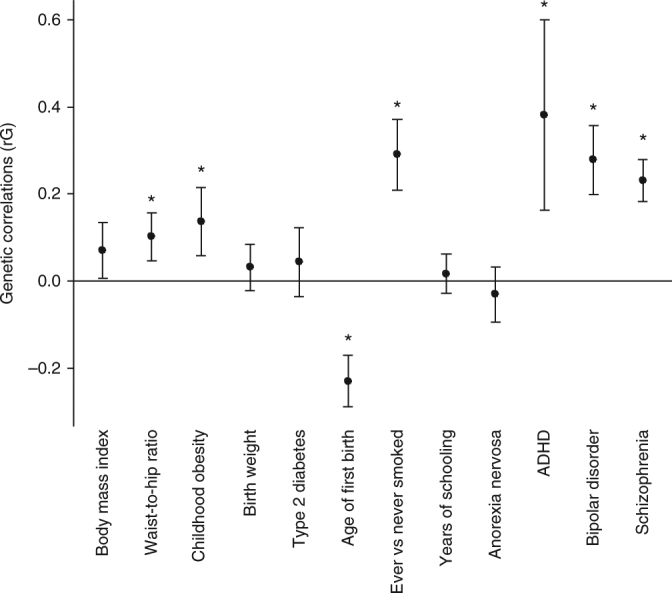
Genetic correlations for risk-taking. Whole-genome LD score regression tested genome-wide SNP associations for risk-taking against similar data for 12 BMI-related traits. Error bars show the 95% confidence intervals for these estimates. Stars indicate statistically significant associations, after adjustment for multiple testing. After correction for multiple testing, WHR and childhood obesity, age at first birth, ever versus never smoking, ADHD, bipolar disorder and schizophrenia remained significant

The genetic correlations between risk-taking propensity and 12 adiposity-related, risk behaviour and psychological traits were calculated using LD score regression. After correction for multiple testing, risk-taking propensity showed positive genetic correlations with: waist-hip ratio (WHR), childhood obesity, ever smoking, ADHD, BPD and schizophrenia; and negative genetic correlations with age at first birth in women (all *P* < 0.004) (Fig. 4).

## Discussion

In this study, we identify 26 genetic loci associated with self-reported risk-taking propensity in a sample of 436,236 participants from the UK Biobank study, 24 of which are novel. Much previous research has focussed on the environmental and psychological determinants of risk-taking behaviours in specific age groups, primarily adolescents, or communities, such as those involved in extreme sports^47–49^. We found several independent genetic signals for risk-taking, which facilitated investigation of its consequences and elucidated the mechanisms of its association with important health outcomes, including obesity, substance abuse and smoking^4,8,50^.

Our findings suggest that the genetic component of risk-taking acts, in large part, through the CNS. We identified four specific brain regions, in addition to the cortex, that show enriched expression for genes associated with risk-taking propensity. All of these regions have previously been implicated in risk-taking-related traits by functional magnetic resonance imaging studies. Activity in the pre-frontal cortex has been linked to behavioural measures of risk-taking^51,52^, the hippocampus has an established role in behavioural inhibition^53^, the anterior cingulate cortex has been implicated in assessing the value associated with exercising control when performing a task^54^, and the hypothalamus is involved in the processing of innate and learned fear, including the fear of pain, predators and aggression^55^. Additionally, enriched expression of risk-associated genes in the immune system supports growing evidence for the influence of the immune system on the brain and human behaviour^56^. While research to date has primarily concerned clinically relevant mood and behavioural aberrations, including depression^57^, an association between immune function and personality has also been proposed^58^.

Genetic correlations between risk-taking and schizophrenia, BPD and ADHD confirm the findings of a smaller, overlapping GWAS of risk-taking among 116,255 UKB participants^29^. Given the genetic and symptomatic overlap between major mental disorders, as well as diagnostic migration and co-segregation within families, traits with trans-diagnostic relevance are important to understanding shared vulnerabilities and mechanisms. Perhaps surprisingly, there was no genetic correlation between risk-taking and years of schooling. Given that the two top hits from this analysis lie within genes involved in cognitive processes (*CADM2* and *FOXP2*), this suggests that there may be a few, specific pleiotropic signals for both traits rather than broader shared mechanisms.

Novel genetic correlations between risk-taking propensity and both childhood obesity and WHR were identified. However, despite previous observed associations between risk-taking and BMI^8,10,11^, we found no genetic correlation between risk-taking and adult BMI. By contrast, the IVW MR analysis links some risk-taking pathways to adult BMI. The high levels of heterogeneity in the MR analysis suggests that the overall genetic correlation (which assumes a linear association between effect sizes for risk-taking and BMI across the genome) may not be the best model to show the complex association between these two traits.

Of the MR analyses performed, only the IVW analysis generated a significant result. While this analysis generally assumes the absence of horizontal pleiotropy, which could not be eliminated here, it has the highest statistical power of the MR analyses performed and is more reliable than the other methods in situations where the effect estimates for associations to the exposure do not vary greatly between SNPs^59^. Our finding of four SNPs with strong associations with both risk-taking and BMI, and with variable directional consistency, suggests that there are diverse pleiotropic pathways that link these two traits.

The complexity of the association between risk-taking propensity and BMI is supported by the directionally inconsistent effects of the four genome-wide significant loci for BMI among the 26 risk-taking propensity-associated loci. The risk-taking increasing variant at three of these four loci is associated with higher BMI, but the risk-taking increasing variant at the remaining signal is associated with lower BMI.

In PRS analysis relating risk-taking propensity to obesogenic behaviours in the Fenland cohort study, we found some suggestion that genetic risk-taking propensity may be associated with emotional eating in men as well as higher daily calorie, fat and protein intake and greater odds of regularly skipping breakfast. These findings require replication in larger data sets. However, they speculatively indicate that obesogenic eating behaviours and practices provide a mechanism through which specific facets of risk-taking propensity are related to BMI.

Risk-taking is a complex phenotype with heterogeneity in methods of measurement between studies and no gold-standard. Studies most often use self-report but behavioural measures are also used. Where studies rely on self-report, questionnaires including multiple items are often used^15^. In the present study, risk-taking propensity was self-reported and based on the answer to a single question that has not previously been used in other studies. Individuals’ responses were stable in the sub-set of participants with repeated measures and self-identification as a risk-taker was associated with risk-taking behaviours, including alcohol consumption, ever smoking, drug addiction and age at first birth, in the anticipated ways. Regardless, research among those involved in extreme sports cautions against assuming psychological or behavioural homogeneity in risk-taking populations^47^. While some risk-takers in these studies may be impulsive, others may take risks in response to feelings of confidence and self-efficacy, justified by experience and the development of expertise^47,60^. The risks that this latter group of individuals are taking are planned, rather than impulsive. The lack of clarifying questions to determine why respondents self-identify as risk-takers is an important limitation of the present study.

The results of the present study advance our understanding of the genetic basis for risk-taking and highlight a common genetic basis for risk-taking and important health-related phenotypes including WHR, depression, schizophrenia and ADHD. Further, the findings indicate the presence of multiple and diverse pathways linking risk-taking to BMI, building on previous observations from non-genetic studies. In particular, these findings suggest a multi-faceted association between risk-taking and BMI, involving pathways that may influence eating and food-related behaviours.

## Methods

### Study populations

*UK Biobank:* The GWAS was conducted among 436,236 white European participants in UK Biobank with both genotype and risk-taking data. UKB is a population-based cohort study of volunteers aged between 40 and 69 years, who were registered with the National Health Service and living within ~25 miles of one of the 22 UKB assessment centres throughout the United Kingdom at the time of recruitment. Recruitment took place between 2006 and 2010. Overall, 503,325 participants were recruited to the cohort after sending invitations to ~9.2 million people^61^. All participants provided written informed consent. The study was approved by the National Research Ethics Service Committee North West–Haydock and all study procedures were performed in accordance with the World Medical Association Declaration of Helsinki ethical principles for medical research.

*The Fenland cohort study:* Eating behaviour, diet and food-related behaviour phenotypes among participants enrolled in the Fenland cohort study were used in the present analysis. The Fenland study is a population-based cohort study of volunteers recruited from participating General Practices in Ely, Wisbech and the surrounding Cambridgeshire region between 2004 and 2015^62^. Eligible individuals were adults registered at a collaborating General Practice and residing in Cambridgeshire at the time of recruitment. Exclusion criteria were: clinically diagnosed diabetes mellitus, inability to walk unaided, terminal illness (life expectancy of ≤1 year at the time of recruitment), clinically diagnosed psychotic disorder, pregnancy or lactation. All participants attended a visit to an MRC Epidemiology Unit testing centre where eating behaviour data was collected. Written informed consent was attained from all participants and the study was approved by the Cambridge Local Research Ethics Committee.

### Phenotypes

*Risk-taking propensity :* As part of the baseline assessment, UKB participants completed a touchscreen questionnaire that included the question *Would you describe yourself as someone who takes risks?* Possible responses were: *Yes*, *No*, *Don’t know* or *Prefer not to say*. A total of 482,173 participants responded either ‘Yes’ (*n* = 129,877) or ‘No’ (*n* = 352,296). Those who answered ‘Don’t know’ or ‘Prefer not to say’ (*n* = 19,538) were excluded from this analysis. During follow-up, the question was asked again to a sub-set of participants. As the sample sizes were substantially decreased between follow-ups, in order to maximise sample size and increase power, we used the baseline responses of all participants for the primary GWAS analysis.

*Eating behaviour:* Eating behaviour was measured in the Fenland cohort using the 18-item version of the Three-Factor Eating Questionnaire (TFEQ-R18)^63^. Three eating behaviours are measured by the questionnaire: CR (6 items), UE (9 items) and EE (3 items). Each item was scored on a 4-point scale (1–4), with higher value indicating more of the behaviour. The items for each of the eating behaviours were then added together and transformed to a 0–100 scale using the following equation: [((raw score − lowest possible raw score)/possible raw score range) × 100]^64^. Scores were  generated on an individual basis, for all Fenland study participants who completed the TFEQ-R18. EE describes a tendency to eat in response to dysphoric emotions, UE indicates a tendency to overeat accompanied by a subjective sense of loss of control over consumption and CR describes the intention to limit food intake in order to influence shape or weight.

A total of 3515 individuals (53.2% women; 98.5% self-reported white ethnicity) aged 35–64 years with intersecting eating behaviour and genotype data were included in the present analysis. Individuals lacking data regarding eating behaviour, age or sex were excluded. The eating behaviour analyses were sex-stratified based on evidence that the behaviours are all significantly higher among women (*P* < 0.0001 for UE and EE; *P* < 0.01 for CR) and reported sex modification of the association between BMI-associated loci and CR^65^.

*Food-related behaviour:* Food-related behaviour was measured as part of the general questionnaire administered to Fenland participants at baseline. To assess snacking while watching television, participants answered: *Apart from meals*, *how often do you snack on foods while watching television?* Possible answers were: *Never or rarely*, *Occasionally*, *Usually*, *Always*. To assess frequency of eating home-cooked meals, participants answered the question: *When you eat your main meal at home*, *how often do you usually eat home cooked meals?* Possible answers were: *Never or less than once a month*, *1*–*2 time per week*, *3*–*5 times per week*, *5+ times per week*. Finally, to assess the frequency of breakfast eating, participants answered: *How often do you usually eat breakfast?* Possible answers were: *Never or less than once a month*, *1*–*2 times per week*, *3*–*5 times per week*, *5+ times per week*.

As the food-related behaviour groups were not continuous, we coded the variables for analysis in logistic regression models. In general, 0 was coded as the more healthy, and 1 as the less healthy response. Frequency of eating home-cooked food was coded: 0 for 5+ times a week; 1 for <5 times a week. Snacking in front of the TV was coded: 0 for never or rarely; 1 for occasionally, usually or always. Frequency of eating breakfast was coded: 0 skips breakfast <2 times a week; 1 skips breakfast ≥2 times a week.

*Dietary information:* Average daily calorie, fat, protein, carbohydrate, fruit, vegetable and fibre intakes were measured using the food frequency questionnaire (FFQ). The FFQ is a validated 130-item semi-quantitative questionnaire that aims to measure self-reported habitual dietary intake over the previous year. Food intake frequency was converted to daily energy (kcal/day) and nutrient intakes (g/day) using FETA 2.53 software^66^. A total of 8981 participants (52.8% women; 98.6% self-reported white ethnicity) aged 30.5–64 years had intersecting genotype, dietary and food-related behaviour data and were included in the present analysis.

### Statistical analysis

*Genotyping*, *imputation and quality control procedures:* We analysed data from the 2017 imputed genetic data, based on the Haplotype Reference Consortium  (HRC) panel release from UKB, comprising 7,736,308 million SNPs. Genotyping, imputation, phasing and quality control are described in detail elsewhere^67^. Briefly, 487,409 of the UKB participants were genotyped using the Affymetrix Applied Biosystems UK Axiom array (Santa Clara, CA, USA), designed to optimise imputation performance in GWAS studies. A small number of participants (*n* = 49,950) were genotyped using the Affymetrix Applied Biosystems UL BiLEVE Axiom Array^68^. The arrays share 95% of their marker content^67^. SNPs were excluded prior to imputation if they were multi-allelic, had missing data or had a minor allele frequency (MAF) < 1%. Phasing was performed using a modified version of the SHAPEIT2 algorithm. Imputation was performed using IMPUTE 2 and a merged reference panel comprised of the 1000 Genomes Project Phase 3 and UK10K haplotype reference panels. In addition to quality control procedures employed by UKB, we defined a white European ancestry set based on a *k*-mean clustering using the first five genetic principle components.

*Genome-wide association analyses:* GWAS testing for associations between SNPs and self-reported risk-taking was performed using a linear mixed model (LMM) implemented in BOLT-LMM^69^. This approach minimises any effect of population structure and permits the inclusion of related individuals in the analysis, maximising statistical power. As all of the top 10 principal components were significantly, but minimally, associated with odds of risk-taking, this approach was appropriate (Supplementary Table 3). SNPs were established based on distance based clumping, using a distance of 1 Mb. Sex, age and genotyping array were included as covariates. SNPs were filtered based on info >0.5 and MAF >1%. Individuals were excluded based on ancestry, withdrawal from the UK Biobank study, mismatch between genetic sex and reported gender and failure of genetic quality control. A total of 436,236 individuals of white European ancestry and 7,736,308 variants were included in the analysis.

Heritability analyses were performed using restricted maximum likelihood implemented in BOLT-LMM, which computes heritability on the observed scale^69^. Genetic variance was calculated for all genotyped autosomal SNPs (*N* = 612,622) for which quality control was performed, adjusting for chip status, age, sex and the top 10 genetically determined principal components. Only unrelated individuals of white European ancestry were included in this analysis (*N* = 339,414).

In the absence of an appropriate data set in which to directly replicate our results, we compared our results in the baseline data set from UK Biobank, to those ascertained using data on risk-taking at the first follow-up assessment and, separately, at the second follow-up assessment. We also conducted a GWAS of a closely related phenotype, ‘ever smoking’, in the same European ancestry UK Biobank sample in order to look up our genome-wide significant SNPs for risk-taking. This sample consisted of 207,229 ever smokers (46%) and 243,177 never smokers.

*Genetic correlations:* Genetic correlations (*r*_g_) were calculated using LD score regression^70^. Genetic correlations between risk-taking and 12 traits available in publicly available data sets were conducted.

*Pathway and tissue enrichment analysis*: We used MAGENTA to implement a gene set enrichment analysis-based approach to test the genome-wide discovery data for associations with biological pathways defined in Go Term, PANTHER, KEGG, Biocarta, Reactome and Ingenuity. MAGENTA maps each gene in the genome to a single index SNP with the lowest *P* value within the window ranging from 110 kb upstream to 40 kb downstream of the gene. This *P* value, representing a gene score, is then corrected in a regression model for confounding factors such as gene size, SNP density and LD-related properties. Each mapped gene in the genome is then ranked by its adjusted gene score. The observed number of gene scores in a given pathway with a ranked score above 75th percentile threshold was calculated. This observed statistic is then compared to one calculated from randomly permuted pathways of identical size. This comparison generates an empirical GSEA *P* value for the pathway. An individual pathway was defined as being significantly enriched when it reached FDR <0.05 in either analysis.

Tissue enrichment analysis was performed using the genotype-tissue expression (GTEx) database^71^. This approach uses stratified LD score regression, a method for partitioning heritability from GWAS summary statistics, to test whether trait heritability is enriched in regions surrounding genes with the highest specific expression in a given tissue^72^. Significance thresholds were established using Bonferroni correction for the number of tests performed.

*Mendelian randomisation:* We conducted a bi-directional MR analysis of risk-taking to BMI using all genome-wide significant variants for risk-taking from the present GWAS. An unpublished GWAS meta-analysis of BMI using UKB plus GIANT data and comprising a total of 772,825 individuals provided effect estimates for BMI. For the risk-taking to BMI analyses, SNPs were aligned to the risk-taking increasing allele. For the BMI to risk-taking MR, SNPs were aligned to the BMI-increasing allele. We used conventional inverse-weighted variance (IVW) MR, by regressing the SNP effect estimates for risk-taking on the SNP effect estimates of the outcome of interest. This analysis was conducted in R version 3.3.1.

As IVW MR assumes the absence of horizontal pleiotropy (heterogeneity) and may be biased by weak instruments, MR Egger and weighted median MR were also performed. The MR Egger method is similar to that of conventional IVW MR. However, unlike IVW MR, the regression is not constrained to pass through the origin. Significant departure of the *y* intercept from zero indicates pleiotropy^73^. The drawback of this method is low statistical power, and susceptibility to bias from weak instruments, which tend to bias results toward the null^59^. Weighted median MR complements MR Egger and allows up to 50% of the information in the MR analysis to come from SNPs that are invalid instruments, including those that are invalid as a result of pleitropy^59^, and yields more precise results than MR Egger if all genetic variants have similar magnitudes of association with the exposure^74^. MR is also limited by factors beyond pleiotropy that cannot be controlled but should be considered. For example, canalisation and compensation might mitigate the effects of genetic changes on outcomes and heterogeneity in exposures may make causal inferences about the dimensions of a trait that are important difficult to infer without biological knowledge.

In order to identify specific SNPs associated with risk-taking that might drive overall effects on BMI, we performed a ‘leave-one-out’ analysis. For this analysis, we repeated the MR of risk-taking to BMI with each of the genome-wide significant SNPs for risk-taking removed, in turn.

*PRS analysis:* A weighted PRS for risk-taking was constructed for Fenland participants (*n* = 11,249) using the summary statistics from the present UKB GWAS. The 26 loci showing genome-wide significant associations with risk-taking were included in the score. At each locus, the number of risk increasing alleles were summed and multiplied by the effect estimate on risk-taking from our UKB GWAS. The results across all 26 SNPs were summed for each participant. The association between the PRS and eating behaviour was examined in the Fenland study using sex-stratified regression models, adjusted for age. The association between the PRS and both the diet and food-related behaviour variables was analysed in Fenland using linear or logistic regression models, as appropriate, adjusted for age and sex. Outcome variables were log-transformed if they were not normally distributed, in order to improve the normality of the residuals.

The following 12 traits were analysed using the PRS: EE, UE, CR, total calorie intake per day, fat intake (g/day), fibre intake (g/day), protein intake (g/day), carbohydrate intake (g/day), fruit and vegetable intake (g/day), snacking while watching TV, frequency of skipping breakfast (times per week) and number of home cooked meals (times per week). This analysis was conducted in Stata version 14.

### Code availability

Code is available upon request from the corresponding authors.

### Data availability

GWAS summary statistics are available at 10.22025/2018.20.202.00002. Individual-level data are available from UK Biobank but restrictions apply to the availability of this data, which was used under license for the current study. Approved researchers may apply for access under the UK Biobank access framework (details can be found here: http://www.ukbiobank.ac.uk/wp-content/uploads/2012/09/Access-Procedures-2011.pdf). Data from the Fenland study are governed in accordance with the *MRC Policy and Guidance on Sharing of Research Data from Population and Patient Studies* and the terms of the participants’ consent and study ethical approvals. Approved researchers wishing to access these data should contact the Fenland Study team (http://epi-meta.medschl.cam.ac.uk/overview.html).

## Electronic supplementary material

Supplementary Information

Description of Additional Supplementary Files

Supplementary Data 1
