# Supplementary material for: Genome–wide association study for risk taking propensity indicates shared pathways with body mass index
Source: Commun Biol. 2018 May 3;1:36. doi: 10.1038/s42003-018-0042-6 (PMC6123697; doi:10.1038/s42003-018-0042-6)
Supplement: Supplementary file 1 — Supplementary Information [file 42003_2018_42_MOESM1_ESM.pdf]

Supplementary Table 1 Look up of genome-wide significant SNPs for risk-taking in UK Biobank for 'Ever smoking'

SNP	Chr	Base pair	Effect allele	Other allele	Effect allele frequency	Info	Beta	SE	*P*-val
rs6762267	3	85513115	C	A	0.381262	0.998347	0.0094076	0.001051	3.40E-19
rs727644	7	114109349	G	A	0.595406	0.992547	0.0013696	0.001045	1.90E-01
rs62519827	8	65481947	T	C	0.887242	1	0.0011878	0.001616	4.60E-01
rs9841382	3	181408124	C	T	0.144996	0.992706	0.0059097	0.001453	4.70E-05
rs58560561	1	243537729	G	T	0.651824	0.985229	-8.50E-05	0.001078	9.40E-01
rs992493	4	106180264	T	C	0.188643	0.99873	0.0049033	0.001307	1.80E-04
rs6923811	6	27289776	T	C	0.678951	1	0.004272	0.001097	9.90E-05
rs7817124	8	81404008	C	G	0.239069	0.997976	0.0022905	0.001199	5.60E-02
rs4801000	18	53456943	G	A	0.33539	0.997479	0.0023003	0.001083	3.40E-02
rs4653015	1	33776431	T	C	0.26086	0.994959	0.0026161	0.001164	2.50E-02
rs12476923	2	145830053	A	C	0.336033	0.999171	-0.0007015	0.001078	5.20E-01
rs283914	3	17330649	T	C	0.531356	0.997244	0.0019071	0.001024	6.20E-02
rs4233093	1	73446245	A	G	0.516179	0.997724	0.0050554	0.001022	7.60E-07
rs7829912	8	33479228	T	C	0.555727	0.991336	0.001943	0.001031	6.00E-02
rs3117340	6	29210596	G	T	0.622344	0.999729	0.0051953	0.001055	8.50E-07
rs1381287	14	98597552	T	C	0.456905	0.986444	0.0050533	0.001032	9.80E-07
rs28520003	22	46411969	G	A	0.68587	1	0.0057744	0.001102	1.60E-07
rs12115650	9	126367705	G	A	0.725133	0.986183	-0.00184	0.001151	1.10E-01
rs11226319	11	104221573	A	G	0.160313	0.994423	-0.0015943	0.001394	2.50E-01
rs1358391	7	115111838	G	T	0.505524	0.986954	0.0050478	0.001027	8.80E-07
rs12617392	2	27336827	C	A	0.558239	0.992674	-0.0003932	0.00103	7.00E-01
rs542883	2	45143382	C	G	0.559253	0.995628	0.0079359	0.001029	1.20E-14
rs10823791	10	73338334	T	A	0.398774	0.997238	0.00158	0.001045	1.30E-01
rs34905321	6	109131107	T	C	0.567814	0.995805	0.0013402	0.001033	1.90E-01
rs891124	16	71440756	T	C	0.710131	0.988667	0.0036719	0.001132	1.20E-03
rs35914833	14	94182383	C	T	0.684169	0.978438	0.000966	0.001111	3.80E-01

The highlighting indicates nominal statistical significance in the ever smoking dataset (P<0.05)

Supplementary Table 2 Associations between four risk-taking loci that are genome-wide significant for BMI and the eating behaviours in the Fenland cohort

Variant	Implicated gene	BMI		Emotional eating		Uncontrolled eating		Cognitive restraint	
		**Beta (SE)**	***P*-val.**	**Beta (SE)**	***P*-val.**	**Beta (SE)**	***P*-val.**	**Beta (SE)**	***P*-val.**
rs891124	CALB2	0.01 (0.002)	3.5x10-10	1.12 (0.71)	0.11	0.48 (0.47)	0.30	0.03 (0.51)	0.96
rs35914833	PRIMA1	0.02 (0.002)	5.3x10-14	0.06 (0.71)	0.93	-0.01 (0.47)	0.98	-0.11 (0.51)	0.83
rs6762267	CADM2	0.02 (0.002)	1.7x10-15	0.95 (0.65)	0.15	0.69 (0.43)	0.11	0.33 (0.47)	0.48
rs7817124	ZBTB10	-0.01 (0.002)	1.8x10-9	0.31 (0.73)	0.67	0.17 (0.48)	0.72	0.37 (0.52)	0.48

SNPs were aligned to the risk taking propensity-increasing allele. Effect estimates (beta and standard error, SE) were derived from linear regressions of the variant to the named trait, adjusted for age and sex. BMI was a continuous outcome standardised within the BMI meta-analysis.

Supplementary Table 3 Associations between the principal components and risk-taking

	**Odds ratio**	**95% CI**	***P*-value**
**PC1**	1.008	(1.005, 1.011)	<0.001
**PC2**	0.995	(0.992, 0.998)	0.002
**PC3**	1.004	(1.000, 1.008)	0.036
**PC4**	0.990	(0.988, 0.992)	<0.001
**PC5**	1.012	(1.010, 1.013)	<0.001
**PC6**	1.004	(1.001, 1.008)	0.013
**PC7**	0.995	(0.992, 0.998)	<0.001
**PC8**	1.009	(1.006, 1.012)	<0.001
**PC9**	0.998	(0.996, 0.999)	0.004
**PC10**	1.003	(1.000, 1.006)	0.037

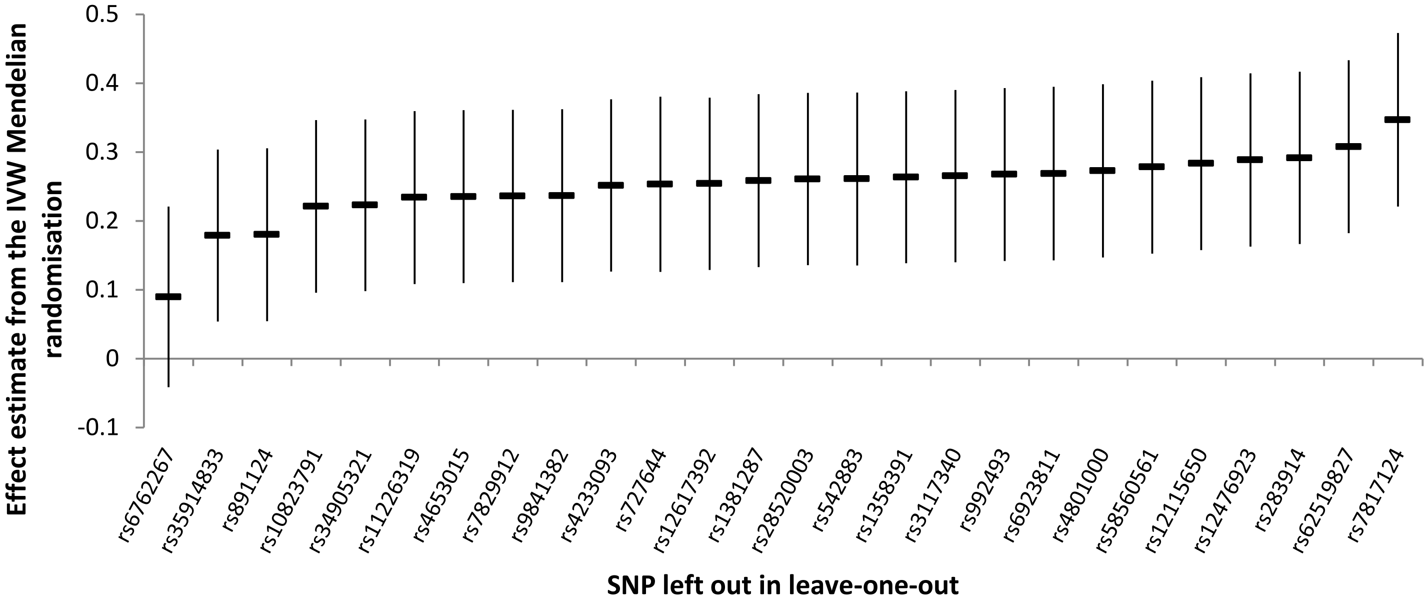

Supplementary Figure 1. Leave-one-out analysis. The figure shows the effect estimates and 95% confidence intervals from a series of inverse weighted variance (IVW) Mendelian randomisation analyses, each with one of the genome wide significant risk-taking propensity SNPs removed. The removed SNP is indicated on the x-axis. Comparison of the effect estimates for the four SNPs that reached genome-wide significance for BMI (rs6762267, rs35914833, rs891124 and rs7817124) with the mean of the effect estimates from the sample with that SNP removed, showed a significant difference ($P<0.0001$).

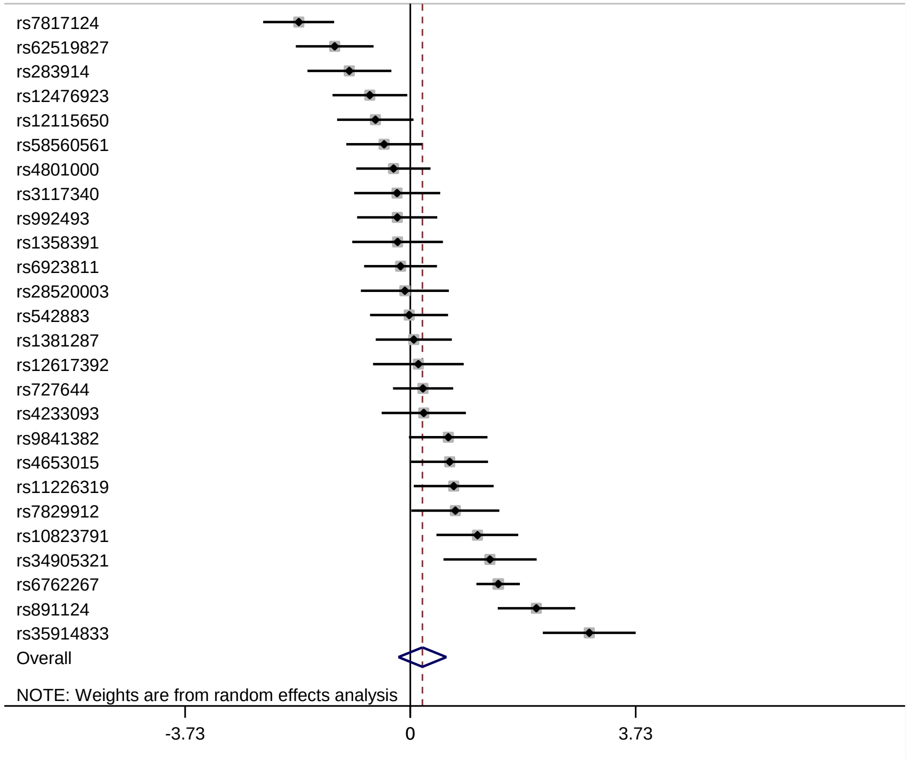

Supplementary Figure 2. Results of a random effects IVW Mendelian randomisation analysis of risk-taking to BMI. This analysis combined the effect estimates ascertained when treating each of the genome-wide significant SNPs for risk-taking (displayed on the y-axis) as an individual instrument. Points indicate effect estimates, bars indicate 95% confidence intervals.

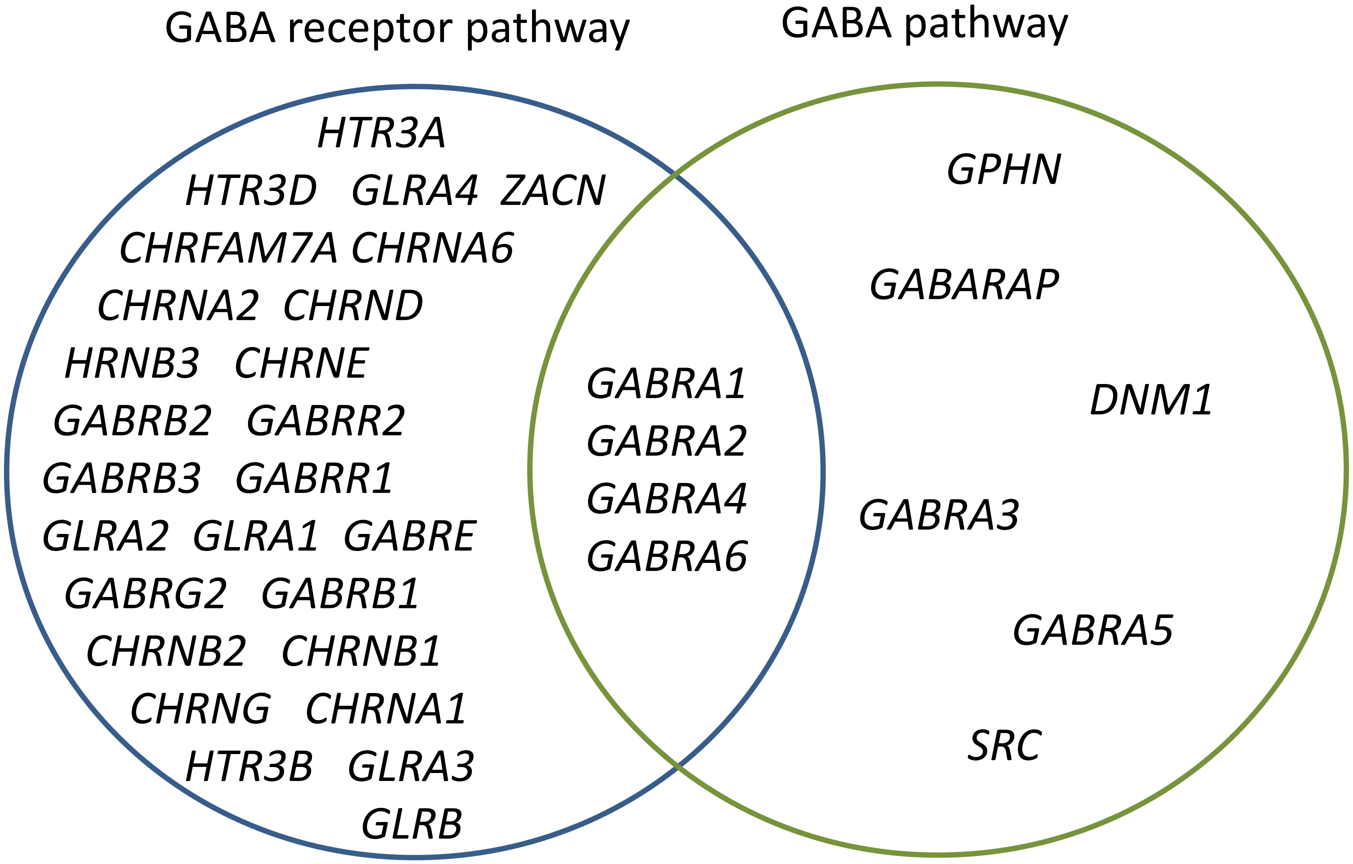

Supplementary Figure 3 Overlap between the GABA pathway and the GABA receptor pathway
