# Supplementary material for: Genome–wide association study for risk taking propensity indicates shared pathways with body mass index
Source: Commun Biol. 2018 May 3;1:36. doi: 10.1038/s42003-018-0042-6 (PMC6123697; doi:10.1038/s42003-018-0042-6)
Supplement: Supplementary file 2 — Description of Additional Supplementary Files [file 42003_2018_42_MOESM2_ESM.docx]

**Description of Additional Supplementary Files**

File Name: Supplementary Data 1

Description: Full MAGENTA output showing the results of tests of all annotated biological pathways for enrichment of genes located near risk-taking propensity associated variants. Two overlapping pathways were identified and are highlighted: the GABA pathway and the GABA receptor pathway.
